# Assessing the Exceptionality of Coloured Motifs in Networks

**DOI:** 10.1186/1687-4153-2009-616234

**Published:** 2008-10-26

**Authors:** Sophie Schbath, Vincent Lacroix, Marie-France Sagot

**Affiliations:** 1 Institut National de la Recherche Agronomique (INRA), UR1077, Unité Mathématique, Informatique et Génome, 78352 Jouy-en-Josas, France; 2 Centre for Genomic Regulation (CRG), Genome Bioinformatics Group, Universitat Pompeu Fabra, Dr. Aiguader 88, 08003 Barcelona, Spain; 3 Université de Lyon, 69000 Lyon, France; 4 Laboratoire de Biométrie et Biologie Évolutive, Université Claude Bernard Lyon 1, CNRS/UMR 5558, 69622 Villeurbanne, France; 5 Projet BAMBOO, Institut National de Recherche Informatique et en Automatique (INRIA) Rhône-Alpes, 655 avenue de l'Europe, 38330 Montbonnot Saint-Martin, France

## Abstract

Various methods have been recently employed to characterise the structure of biological networks. In particular, the concept of network motif and the related one of coloured motif have proven useful to model the notion of a functional/evolutionary building block. However, algorithms that enumerate all the motifs of a network may produce a very large output, and methods to decide which motifs should be selected for downstream analysis are needed. A widely used method is to assess if the motif is exceptional, that is, over- or under-represented with respect to a null hypothesis. Much effort has been put in the last thirty years to derive -values for the frequencies of topological motifs, that is, fixed subgraphs. They rely either on (compound) Poisson and Gaussian approximations for the motif count distribution in Erdös-Rényi random graphs or on simulations in other models. We focus on a different definition of graph motifs that corresponds to coloured motifs. A coloured motif is a connected subgraph with fixed vertex colours but unspecified topology. Our work is the first analytical attempt to assess the exceptionality of coloured motifs in networks without any simulation. We first establish analytical formulae for the mean and the variance of the count of a coloured motif in an Erdös-Rényi random graph model. Using simulations under this model, we further show that a Pólya-Aeppli distribution better approximates the distribution of the motif count compared to Gaussian or Poisson distributions. The Pólya-Aeppli distribution, and more generally the compound Poisson distributions, are indeed well designed to model counts of clumping events. Altogether, these results enable to derive a -value for a coloured motif, without spending time on simulations.

## 1. Introduction

Descriptions of biological networks serve two main purposes. On the one hand, it enables to address questions related to the evolution of the network, that is, how such a complex structure has been set up in the course of evolution. On the other hand, structural analysis can be seen as a first necessary step prior to a dynamical analysis which in turn enables to simulate networks and to study their response to perturbation. Usually, three main classes of biological networks are considered [1]: protein interaction, gene regulatory, and metabolic. When analysing their structure, these networks are usually modelled as graphs, where vertices represent molecules (metabolites, genes, and proteins) and edges (directed or undirected) represent interactions between these molecules (the direction, when it is known, indicating which molecule is acting upon the other). For instance, in the case of a gene regulatory network, vertices correspond to genes and there is a directed edge from a gene coding for a transcription factor to every gene that this transcription factor regulates.

The structure of a biological network may be apprehended by using a variety of measures, such as vertex degree [2], degree correlation [3], or average shortest path length [4].

In this paper, we focus on the concept of motif. A network motif has been initially defined as a pattern of interconnections which occurs unexpectedly often in a network [5, 6]. The assumption generally made is that subnetworks sharing the same topology will be functionally similar. Over- (resp., under-) represented subnetworks may therefore correspond to conserved (resp., avoided) and thus important (resp., vital/detrimental) cellular functions. In the context of regulatory networks, simple patterns such as loops may be interpreted as logical circuits controlling the dynamic behaviour of a network. If the over- and under-representations of network motifs are often assessed via simulations of random networks in practice, approximations of the subgraph count distribution in various random graph models have been proposed in the literature. Some of these approximations can be found in the book by Janson et al. [7] or in more recent studies such as those by Stark [8], Itzkovitz et al. [9], Camacho et al. [10], and Picard et al. [11].

**Figure 1:**
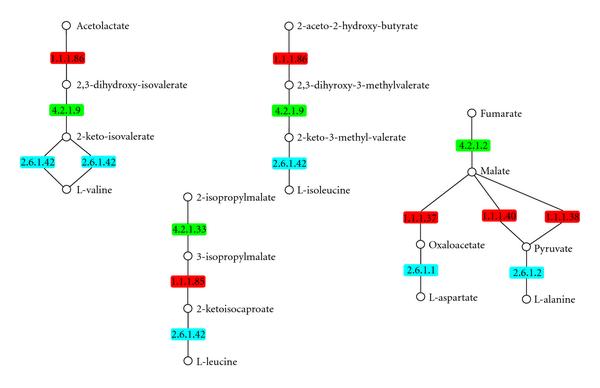
Similar sets of transformations in the metabolic network of the bacterium *Escherichia coli*.

A limitation of the notion of topological motif is that in many cases the same subgraph may in fact correspond to different functions, depending on the nature of the vertices that compose it. This is typically the case for metabolic networks whose fullest representation is in terms of a bipartite graph with two sets of vertices, one corresponding to reactions and the other to chemical compounds, those reactions are required as input or produced as output. Topological motifs which neglect vertex labels (for the reactions and/or the compounds) may associate completely different chemical transformations, while motifs that took such labels into account but enforced topological isomorphism would miss the fact that some sets of similar transformations may occur in different order. A biological example of the latter is given in the simple case of linear sets of transformations in Figure 1, where rectangles are reactions and circles are compounds. More complex examples are discussed in Lacroix et al. [12].

Moreover, in some situations, as, for example, in the case of protein interaction networks, the topology of the network is not fully known. Indeed, high-throughput experiments used to obtain large-scale protein interaction data are notoriously noisy, that is, they may detect interactions when there is none (false positive) and they may miss existing interactions (false negative). In this context, it may be inadequate to look for exact repetitions of a pattern. An alternative definition has thus been proposed, where a motif is defined by using the labels of its vertices and only connectedness of the induced subgraph is required [12].

A coloured motif is defined as a *multiset* of colours (vertex labels), that is, a motif may contain colours whose multiplicity are greater than 1. The cardinality of a motif, that is, of the multiset, will be called the size of a motif. An occurrence of a motif is defined as a connected subgraph whose labels match the motif.

The enumeration of coloured motifs is a nontrivial task which has been the subject of several works [12, 13] which allowed to establish the complexity of the problem and provide algorithms to efficiently detect all the occurrences of a motif in a graph. In practice, current methods now allow to enumerate all the motifs of size 7 of a graph representing the metabolic network of a bacterium in less than two hours. Beyond the time complexity of the task, a major challenge that remains open is to make sense of the potentially very large output of such an enumeration procedure, especially when the focus is not on a single motif but on all motifs of a given size. Ideally, one would need a method to rank the motifs according to their biological relevance in order to prioritise a small number of motifs for downstream analysis. However, the notion of biological relevance is generally ill defined, and a classically used approximation is its statistical significance (or exceptionality).

The exceptionality of a coloured motif, that is the over- or under-representation of the motif with respect to a null model, can be assessed by comparing the observed count of occurrences of a motif to the expected count of the same motif under a null hypothesis. Up to now, this procedure was performed (e.g., in MOTUS [14], http://pbil.univ-lyon1.fr/software/motus/) using simulations: a large number of random graphs were generated and the motif of interest was sought in each one, generating an empirical distribution of the motif count to which the observed count could be compared in order to derive a -score and a -value. The main limitation of this procedure is that it adds a multiplicative factor to the time complexity of the algorithm. Moreover, it is not trivial to choose the optimal number of simulations to perform in order to get a satisfactory estimation of the -value. As a rule of thumb, in order to estimate quite accurately a -value of 1 over , at least  simulations should be performed.

In this paper, we propose a new approach for assessing the exceptionality of coloured motifs which do not require simulations and therefore circumvents the previously mentioned limitations. We were able to establish exact analytical formulae for the mean and the variance of the count of a coloured motif in an Erdös-Rényi (ER) random graph model. Thanks to these results, one can now derive a -score for each motif and therefore rank them according to their exceptionality. We then worked on modelling the complete distribution of the count of a coloured motif in an ER random graph model. To this purpose, we performed a large number of simulations, using different colour frequencies for the motif and different number of vertices and edges for the graph. We could establish that the Poisson distribution was not appropriate whereas the Pólya-Aeppli distribution was a good and better approximation than the commonly used Gaussian distribution. The choice of a Pólya-Aeppli distribution was driven by the following facts: (i) motif occurrences overlap in a network, as shown in Figure 1; (ii) compound Poisson distributions are particularly adapted to model counts of clumping events [15, Chapter 9]; (iii) Pólya-Aeppli approximations are efficient for the count of words in letter sequences [16]. These results can in turn be used to derive a -value for each motif, and, therefore, to introduce a cut-off for deciding which motifs should be selected for downstream analysis.

To our knowledge, there has been no previous work on the significance of coloured motifs in random graphs. This is the reason why we started by focusing on the more general random graph model that is available. We are aware that this may not be the most suitable model to describe the structure of a biological network. However, we argue that this work provides a first necessary basis which can later be extended to richer models, such as the promising mixture of Erdös-Rényi models proposed by Daudin et al. [17].

## 2. Definitions and Notations

Coloured Random Graph Model

We consider a random graph  with  vertices . We assume that random edges are independent and distributed according to a Bernoulli distribution with parameter  (the so-called Erdös-Rényi model). Moreover, vertices are randomly and independently coloured as follows. Let  be a finite set of  different colours and  a probability measure on :  is then the probability for a vertex to be coloured with .

In a metabolic network, the colours of reaction vertices can represent classes of chemical transformations; in regulation networks, the colours of gene vertices can represent functional classes. For defining these classes, the EC number hierarchy (http://www.chem.qmul.ac.uk/iubmb/enzyme/) or Gene Ontology (http://www.geneontology.org/GO.doc.shtml) is classically used.

Coloured Motif

We consider motifs as introduced in Lacroix et al. [12]: a (coloured) motif  of size  is a multiset of  colours . Colours from a motif may not be different, that is, one may have  for some . We then denote by  the multiplicity of the colour  in . When there is no ambiguity,  will simply be denoted by . The notion of multiplicity of a single colour in  will be extended to a multiset of colours in Section 3.2.

Motif Occurrences

**Figure 2:**
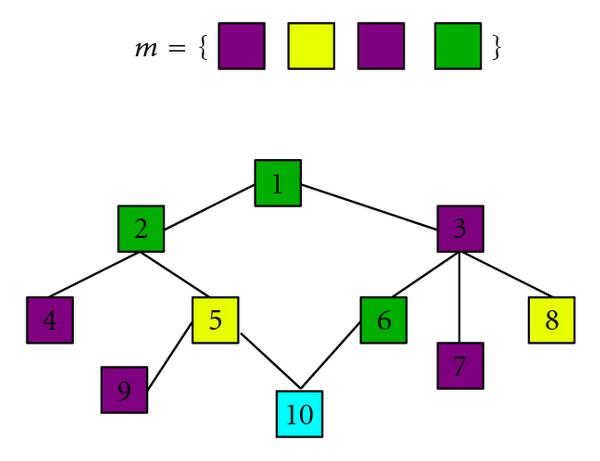
**Example of a graph and a motif.** The motif  occurs three times in the graph, at positions  and .

We now define an occurrence of such a coloured motif. To this purpose, we introduce the following notation. If  are  different indices from  then  represents the subgraph of  induced by the vertices . Let  be the set of all the subsets of size  from . We say that a motif  occurs at position  if and only if  is connected and the colours of , denoted by , are exactly .  corresponds, then, to the set of possible positions for the occurrence of a motif of size . Figure 2 gives an example of a motif and its occurrences.

Number of Occurrences

We introduce the random indicator variable  which equals one if motif  occurs at position  in  and zero, otherwise(1)

where  is then a Bernoulli random variable whose expectation is denoted by : (2)

The probability  for  to occur at position  will be given in Section 3.1.

The number of occurrences of the motif  in the graph , denoted by , is defined by(3)

## 3. Mean and Variance for the Count

This section will provide analytical formulae for the mean and the variance of the number of occurrences of a coloured motif in a random graph. It involves the computation of some probabilities of connectedness. The generalisation to the number of occurrences of a set a coloured motifs will be done in the supplementary material.

### 3.1. Mean Number of Occurrences

The mean number of occurrences of the motif  in the graph  simply follows from the count expression (3):(4)

where  is the occurrence probability of the motif and is given below by (6).

Occurrence Probability

The probability  for  to occur at position  is simply equal to the product of two probabilities: the probability that  is connected and the probability to assign colours  to vertices . The latter, denoted by , follows from the multinomial distribution(5)

leading to(6)

where  denotes the probability for a random graph (Erdös-Rényi model) with  vertices and edge probability  to be connected (by definition, ).

Connectivity Probability

The probability  is calculated recursively [18] as follows:(7)

where . For instance, for , which is typically the range for the motif size in practice, we have(8)

### 3.2. Variance of the Number of Occurrences

Getting the variance is much more involved. We start from  and we have to compute the moment of order two(9)

First, the sums over  and  are calculated according to the number  of vertices shared by the subgraphs  and : (10)

Second, we use the fact that  and  are indicator variables which lead to . These random variables are not independent but the above probability can be written as(11)

with(12)

The terms  and  are now separately calculated.

Computation of 

Let ; the subgraphs  and  have thus  vertices in common, with . Let  such that  and denote ;  represents the colours of the  vertices shared by  and . The multiplicity of colour  in  (resp., in ) is denoted by  (resp., ). To calculate , we start by choosing the  colours  of  (event with probability ), then the  remaining colours  are spread over both  (event with probability ) and  (event with probability ). Finally, one just has to sum over all possible *different* which is equivalent to summing over all  and dividing each term by the multiplicity of  in . This leads to(13)

where  is the multiplicity of  in . For instance, if ,  and  then the multiplicity of  in  equals 2 whereas the multiplicity of  equals 1.

Computation of 

Let again . If  (i.e.,  and  are disjoint) or  (i.e.,  and  have a unique vertex in common) then the events  and  are independent leading to(14)

Another easy case is when  because it means that  and therefore(15)

For the other cases, no general formulae have been found so far but for small values of  one can automatically enumerate all the solutions thanks to the edge binary tree, as described below. As an illustration, the case  (and ) will be detailed.

**Figure 3:**
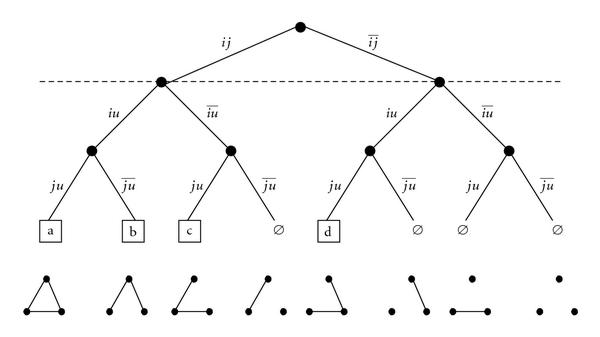
**Complete edge binary tree for vertices ,  and .** Branches are labelled according to the presence or absence of edges: label  for instance, means that  and  are connected, whereas  means the opposite. Leafs which correspond to connected subgraphs are represented by a square.

The principle is to work conditionally to the subgraph (16)

where  is any subgraph of  vertices. Since  is typically small, both probabilities can be computed by enumerating all possible subgraphs  and . This can be done by traversing the complete edge binary tree associated to the  potential edges of  that is, to the binary tree whose branches are labelled according to the presence or absence of edges in the subgraph . This tree is composed of  levels, one for each potential edge and each internal vertex in this tree has two sons: the left one corresponds to the presence of the corresponding edge in the graph whereas the right one corresponds to its absence. It follows that each path from the root to a leaf corresponds to one of the  possible graphs of size . Figure 3 gives an example for . Vertices are labelled , the higher level corresponds to the edge , the middle one corresponds to the edge  and the lower level corresponds to the edge . Leaves corresponding to connected graphs are drawn with a square. In practice, the connectedness of a graph can be checked thanks to its adjacency matrix to the power . Indeed, a graph of size  with adjacency matrix  is connected if and only if  contains no zero (every vertex can be reached from any vertex in at most  steps). Additionally, the binary tree is built such that all pairs of common vertices between  and  are at the top levels. The probability of each connected graph of size  can then be easily calculated when traversing the tree and likewise for both probabilities appearing in (16).

As an illustration, we now detail the computation for  and . Let  and  be the two common vertices between  and , and let  be the third vertex of  (). The edge binary tree is given by Figure 3. In this case, there are only two subgraphs  with  vertices: either  and  are connected (probability ) or they are not connected (probability ). In Figure 3, we indicate with a dashed horizontal line the separation between edges in  (the conditioning event) and edges in . Overall, with , there are four possible connected subgraphs : the triangle (labelled by "a") and the three possible "Vs" (labelled by "b", "c", and "d"). The probability that  is connected given  is obtained from cases "a" (probability ), "b" (probability ), and "c" (probability )(17)

The probability that  is connected given that  is not connected with  is obtained from case "d" (probability ), leading to(18)

Using this algorithm, we find the following results for  and  ( can be processed with the trivial formulae (14) or (15)):(19)

Finally, we obtained analytical formulae for the variance.

## 4. Towards the Motif Count Distribution: A Simulated Approach

Aim

No theoretical results exist so far on the distribution of coloured motifs in random graphs. In this paper, we propose an approximation for this distribution. Thanks to simulations, we first studied the quality of the normal approximation which is classically assumed, especially when using -scores [5, 12]. However, network motif occurrences tend to overlap in networks. It is well known from probability theory that compound Poisson distributions are more relevant than Gaussian distributions to model the count of rare and clumping events. Besides, a compound Poisson approximation for the count of particular subgraphs (topological network motifs) has been proposed by Stark [8] under certain asymptotic conditions on the ER random graph model. Moreover, by analogy with pattern occurrences in letter sequences [16], Picard et al. [11] recently investigated a particular compound Poisson approximation, namely, a Pólya-Aeppli approximation, and concluded that this distribution fits well the count of topological network motifs. The Pólya-Aeppli distribution (denoted by ) with parameters  is the distribution of , where the number of clumps  is Poisson distributed () and the size  of the clumps is geometrically distributed (). Its mean is equal to  and its variance equals . We have then also considered the Pólya-Aeppli approximation. We did not investigate the Poisson approximation because, as we can see on Table 1, the variance of the count (whatever the coloured motif) is quite different from the mean count.

**Table 1:** Quality of approximation of the count distribution for  and . The empirical mean , variance , and cumulative distribution function  have been obtained thanks to 10 000 random graphs.  are the parameters of the Pólya-Aeppli distribution.  and  are the Kolmogorov-Smirnov distances. For  then ,  is the  quantile of the normal distribution (idem for the Pólya-Aeppli distribution).

									****				****			
**Motif **	****	****	****	****	****	****	** (%)**	** (%)**	****	** (%)**	****	** (%)**	****	** (%)**	****	** (%)**
111	1023.65	27462.66	1021.97	27446.53	0.93	73.37	2.40	0.78	1407.4	1.6	1436	1.1	1533.9	0.23	1591	0.12
122	767.74	14941.43	766.05	14660.79	0.90	76.08	2.14	0.65	1047.7	1.5	1068	1.0	1140.2	0.25	1181	0.07
123	614.19	8546.68	615.26	8493.22	0.86	83.12	1.75	0.68	829.6	1.4	845	0.8	900.0	0.18	929	0.08
114	307.09	5729.89	307.77	5807.09	0.90	30.98	3.20	0.71	485.0	1.5	505	0.8	543.3	0.28	583	0.08
134	122.84	1305.02	123.06	1311.64	0.83	21.11	3.43	0.78	207.3	1.8	219	0.9	235.0	0.37	257	0.12
115	61.41	1180.68	61.72	1147.95	0.90	6.30	5.72	0.98	140.5	2.3	160	0.8	166.4	0.57	205	0.06
244	15.35	85.99	15.29	85.57	0.70	4.63	8.73	1.07	36.8	2.4	43	0.8	43.9	0.81	55	0.12
245	6.14	27.76	6.20	28.45	0.64	2.22	12.72	1.27	18.6	2.5	23	0.8	22.7	1.09	32	0.10
345	2.46	6.63	2.51	6.58	0.45	1.39	17.97	0.53	8.5	1.9	11	0.5	10.4	0.77	15	0.09
155	1.23	6.94	1.22	6.74	0.69	0.37	34.23	5.75	7.2	3.3	12	0.6	9.2	1.56	20	0.05
444	1.02	2.46	1.02	2.51	0.42	0.59	27.39	3.80	4.7	2.4	7	0.5	5.9	1.48	10	0.09
355	0.25	0.50	0.25	0.50	0.34	0.16	48.47	0.43	1.9	2.5	3	0.4	2.4	0.96	6	2e-05
455	0.12	0.20	0.13	0.20	0.23	0.09	51.63	0.16	1.2	0.6	2	0.1	1.5	0.65	4	0.03
555	0.008	0.01	0.007	0.008	0.035	0.007	52.61	2e-03	0.2	0.03	0	0.03	0.3	0.03	1	2e-05

Simulation Design

We have simulated 10 000 Erdös-Rényi random graphs with  vertices () and edge probability . Vertices have been randomly coloured with 5 colours () and according to the following colour frequencies: . These choices for , , and  allow to get coloured motifs of size 3 with a wide range of expected counts. We have then selected 14 motifs of size 3 to cover both this variety of counts and different multiplicity pattern: , , , , , , , , , , , ,  and .

**Figure 4:**
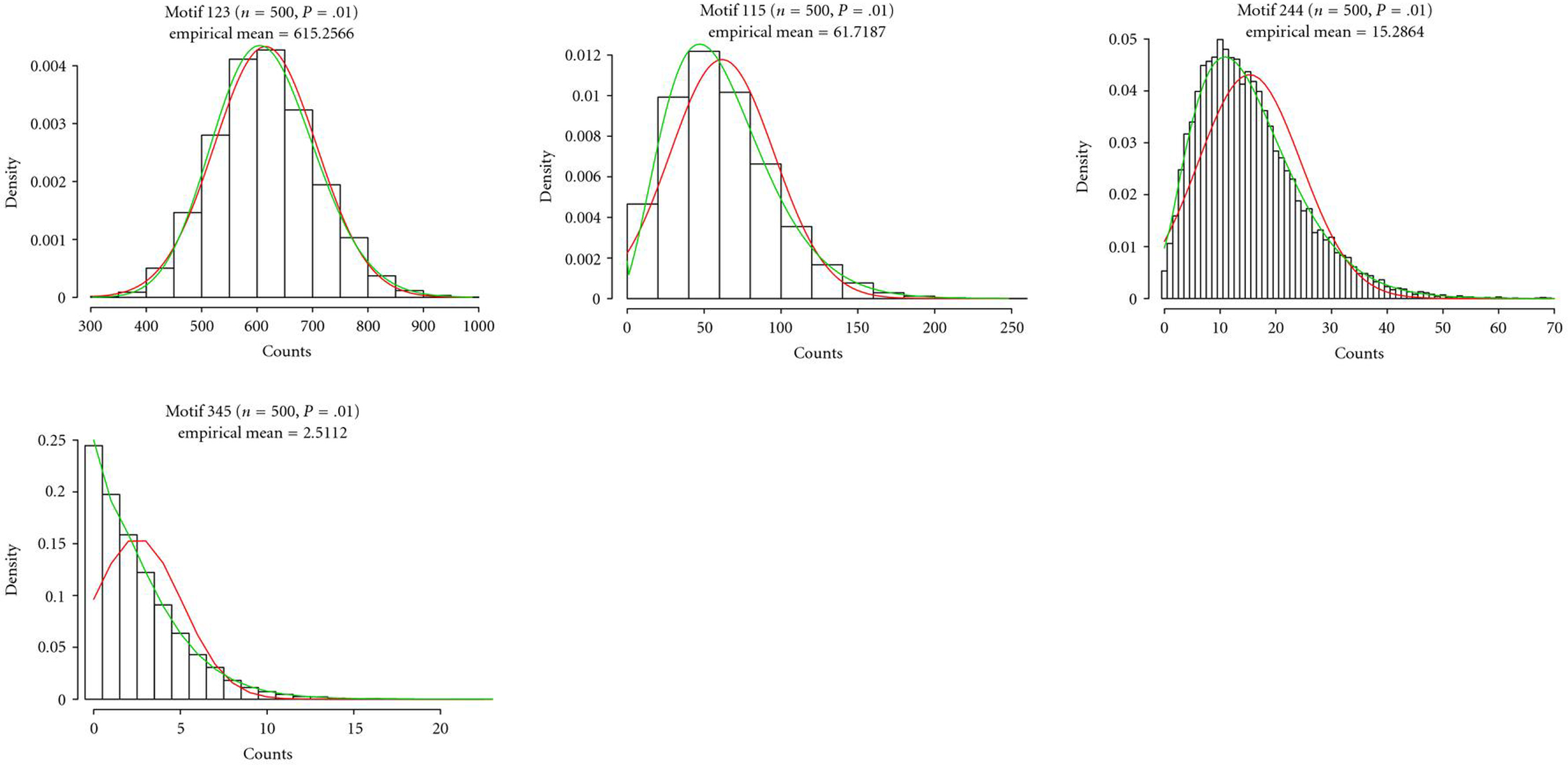
**Empirical distributions for the count of motifs , ,  and  in random graphs with  and .** The empirical means are, respectively, 615, 61, 15, and 2. The red (resp., green) curves correspond to the ad hoc normal distributions (resp., Pólya-Aeppli distributions).

For each motif and each couple , we then obtained an empirical distribution which has been compared with both the normal distribution  and the Pólya-Aeppli distribution  with  and  (see Figure 4 for 4 representative examples).

Quality of Approximation

To measure this quality, we adopted two criteria: (1) the Kolmogorov-Smirnov distance which measures the maximal difference between the empirical cumulative distribution function (cdf)  and the cdf of the normal or the Pólya-Aeppli distribution. The closer to 0 the KS distance, the better the approximation. (2) 1 minus the empirical cdf calculated at the  and  quantiles of the normal or of the Pólya-Aeppli distribution. The closer to 1% and 0.1% these values, the better the approximation.

Results

Results for different values of  and  are very similar. We only present here the ones corresponding to  and  because these values are very close to those observed in real cases such as the metabolic network of *E. coli* as considered in Lacroix et al. [12]. Nevertheless, all results are presented in the supplementary material.

We can first notice just by eye (see Figure 4) that the normal distribution seems satisfactory for frequent motifs but the rarer the motif, the worse the goodness-of-fit. The Pólya-Aeppli distribution seems to fit quite correctly the count distribution whatever the motif. These initial impressions are emphasised when we look at the Kolmogorov-Smirnov distances (see Table 1). The ones for the Pólya-Aeppli distribution are always smaller than those for the normal distribution and sometimes much smaller. In fact, the distance to the normal distribution is quite large for very rare motifs (typically when ). If we now concentrate on the distribution tails by looking at the empirical probabilities to exceed the 99% or 99.9% quantiles  and , we can also notice that they are closer to 1% or 0.1% for the Pólya-Aeppli distribution than for the normal distribution. For extremely rare motifs, quantiles  for both 99% and 99.9% could not be correctly calculated because the corresponding Pólya-Aeppli distribution is both discrete and concentrated around 0. The values for the empirical tails provided in the table are therefore not meaningful in such cases, but thanks to the very small KS distances, we can check that the approximation is still good. Finally, observe that most of the time the normal distribution underestimates the quantile (the empirical right tail is overestimated) leading to false positives.

## 5. Discussion and Conclusion

In this paper, we proposed a new way to assess the exceptionality of coloured motifs in networks which do not require to perform simulations. Indeed, we were able to establish analytical formulae for the mean and the variance of the count of a coloured motif in an Erdös-Rényi random graph model. Furthermore, using simulations, we showed that the motif count distribution can be quite accurately approximated with a Pólya-Aeppli distribution, and that neither the Gaussian nor the Poisson distributions are relevant. Altogether, these results now allow to derive a -value for a coloured motif without performing simulations. Clearly, when several motifs have to be tested, which is the case in the context of motif discovery, one has to control for multiple testing. A conservative strategy that is classically used and that we would recommend is then to apply a Bonferroni correction.

In this work, we did not investigate the case of long motifs, but we can anticipate that motifs containing submotifs which are exceptional will tend to be exceptional themselves. This type of phenomenon is also observed for patterns in sequences and a classical way to deal with it is to control for the number of sequence patterns of size  (by using a Markov model of order ), when assessing the exceptionality of patterns of size . However, in the case of networks, the problem is far from trivial and it is unclear, even for small values of  if the space of random graphs verifying these constraints will not be too small. In the worst case, this space may even be reduced to the observed graph itself.

Also in the case of very rare motifs, the expected distribution of the count is essentially concentrated around 0. Therefore, a single occurrence of such a motif will often be sufficient for it to be considered as exceptional. If we now consider the extreme case of a coloured graph, where each vertex is assigned a different colour, then all possible motifs will be very rare and, therefore, they may all be detected as exceptional. In practical cases, such as for the network representing the metabolic network of the bacterium *E. coli*, the situation is less dramatic but indeed many colours are present only once. This issue may be partially addressed by considering a random graph model, where the colours and the topology are not independent anymore. This would allow to discriminate between infrequent poorly connected colours and infrequent highly connected colours. Motifs containing the latter type of colours would be expected to have more occurrences and should therefore not be systematically considered as exceptional when they have a single occurrence.

More generally, we considered in this paper a very simple random graph model. Even though we think this work was necessary to establish a framework for accessing the exceptionality of coloured motifs, an important step is now to extend these results to other models of random graphs which better represent the structure of real networks. Different types of models have been proposed in the literature for this purpose, for instance, small-world networks, scale-free networks, preferential attachment models, and fixed degree distribution models. However, these models do not provide the probabilistic distribution on edges which is required to compute the occurrence probability of a motif and the probability of two nondisjoint occurrences. Moreover, it has been shown that subnetworks of scale-free networks lose the scale-free property [19]. This is a real drawback for modelling biological networks because they usually correspond to the partial knowledge we have of a system and are therefore far from complete. An interesting issue would be to generalise our work to a mixture of ER random graph models. These models seem indeed very flexible and are able to fit nicely biological networks [17].

Finally, we think there is still room for improvement on the approximation of the motif count distribution. Indeed, no theoretical evidence has been found so far supporting the use of a geometric distribution for the clump size. Analytically, getting the third moment and eventually the fourth moment of the count could certainly allow to investigate other distributions.

## References

[B1] Alm E Arkin AP Biological networks Current Opinion in Structural Biology 2003 13 2 193 202 10.1016/S0959-440X(03)00031-9 12727512

[B2] Jeong H Tombor B Albert R Oltvai ZN Barabási A-L The large-scale organization of metabolic networks Nature 2000 407 6804 651 654 10.1038/35036627 11034217

[B3] Maslov S Sneppen K Specificity and stability in topology of protein networks Science 2002 296 5569 910 913 10.1126/science.1065103 11988575

[B4] Wagner A Fell DA The small world inside large metabolic networks Proceedings of the Royal Society B 2001 268 1478 1803 1810 10.1098/rspb.2001.1711 11522199 PMC1088812

[B5] Milo R Shen-Orr SS Itzkovitz S Kashtan N Chklovskii D Alon U Network motifs: simple building blocks of complex networks Science 2002 298 5594 824 827 10.1126/science.298.5594.824 12399590

[B6] Shen-Orr SS Milo R Mangan S Alon U Network motifs in the transcriptional regulation network of *Escherichia coli* Nature Genetics 2002 31 1 64 68 10.1038/ng881 11967538

[B7] Janson S Łuczak T Ruciński A Random Graphs 2000 Wiley-Interscience, New York, NY, USA

[B8] Stark D Compound Poisson approximations of subgraph counts in random graphs Random Structures & Algorithms 2001 18 1 39 60 10.1002/1098-2418(200101)18:1<39::AID-RSA4>3.0.CO;2-B

[B9] Itzkovitz S Milo R Kashtan N Ziv G Alon U Subgraphs in random networks Physical Review E 2003 68 2 8 10.1103/PhysRevE.68.026127 14525069

[B10] Camacho J Stouffer DB Amaral LAN Quantitative analysis of the local structure of food webs Journal of Theoretical Biology 2007 246 2 260 268 10.1016/j.jtbi.2006.12.036 17292921 PMC2128744

[B11] Picard F Daudin J-J Koskas M Schbath S Robin S Assessing the exceptionality of network motifs Journal of Computational Biology 2008 15 1 1 20 10.1089/cmb.2007.0137 18257674

[B12] Lacroix V Fernandes CG Sagot M-F Motif search in graphs: application to metabolic networks IEEE/ACM Transactions on Computational Biology and Bioinformatics 2006 3 4 360 368 10.1109/TCBB.2006.55 17085845

[B13] Fellows MR Fertin G Hermelin D Vialette S Sharp tractability borderlines for finding connected motifs in vertex-colored graphs Proceedings of the 34th International Colloquium on Automata, Languages and Programming (ICALP '07), July 2007, Wroclaw, Poland, Lecture Notes in Computer Science 4596 340 351

[B14] Lacroix V Cottret L Rogier O Fernandes C Jourdan F Sagot M-F Motus: a software and a webserver for the search and enumeration of node-labelled connected subgraphs in biological networks submitted

[B15] Johnson NL Kotz S Kemp AW Univariate Discrete Distributions 1992 John Wiley & Sons, New York, NY, USA

[B16] Schbath S Compound Poisson approximation of word counts in DNA sequences ESAIM: Probability and Statistics 1995 1 1 16 10.1051/ps:1997100

[B17] Daudin J-J Picard F Robin S A mixture model for random graphs Statistics and Computing 2008 18 2 173 183 10.1007/s11222-007-9046-7

[B18] Gilbert EN Random graphs The Annals of Mathematical Statistics 1959 30 4 1141 1144 10.1214/aoms/1177706098

[B19] Stumpf MPH Wiuf C May RM Subnets of scale-free networks are not scale-free: sampling properties of networks Proceedings of the National Academy of Sciences of the United States of America 2005 102 12 4221 4224 10.1073/pnas.0501179102 15767579 PMC555505

